# Response to Ceftriaxone in Asymptomatic Neurosyphilis Refractory to Doxycycline and Benzathine Penicillin

**DOI:** 10.7759/cureus.55196

**Published:** 2024-02-28

**Authors:** Kshitiz Lakhey, Ajay Kumar, Rohan Manoj, Namratha B Puttur, Nishtha Malik

**Affiliations:** 1 Dermatology, Venereology and Leprosy, Dr. D. Y. Patil Medical College, Hospital & Research Centre, Dr. D. Y. Patil Vidyapeeth (Deemed to be University), Pune, IND

**Keywords:** failure of oral doxycycline treatment, benzathine penicillin g, plwhiv, ceftriaxone, refractory neurosyphilis, asymptomatic neurosyphilis, neurosyphilis

## Abstract

An asymptomatic male in his mid-30s presented with a positive Venereal Disease Research Laboratory (VDRL) test report. He was investigated and detected to be reactive for human immunodeficiency virus (HIV)-1 antibodies. A lumbar puncture revealed cerebrospinal fluid (CSF) VDRL to be reactive at a titer of 1:160 which led to a diagnosis of asymptomatic neurosyphilis. The unavailability of first-line antibiotics necessitated the search for alternative regimens. The patient was administered oral doxycycline 200 mg twice daily for 28 days along with intramuscular benzathine penicillin 2.4 million units once weekly for three weeks. A repeat CSF-VDRL performed six months later with raised titers of 1:320 indicated treatment failure. The patient was then administered ceftriaxone 1 g intramuscularly for 14 consecutive days. A final CSF-VDRL examination performed six months later showed non-reactive titers.

## Introduction

Neural invasion by treponemes occurs in up to 50% of early infection cases [1]. Those with cerebrospinal fluid (CSF) abnormalities such as reactive CSF Venereal Disease Research Laboratory (VDRL) elevated protein concentration and/or pleocytosis without any neurological symptoms are considered to have asymptomatic neurosyphilis [2]. These CSF abnormalities resolve with the treatment recommended for early syphilis [1].

The recommended regimen for neurosyphilis is intravenous aqueous crystalline penicillin G 18-24 million units per day or intramuscular procaine penicillin G 2.4 million units once daily along with probenecid 500 mg taken per oral four times a day for 10-14 days [3,4]. The addition of benzathine penicillin to provide a treatment duration equivalent to that of latent syphilis is also recommended [3]. Two alternate regimens recommended include oral doxycycline 200 mg per oral twice daily [4] for 28 days or 1-2 g ceftriaxone intramuscularly for 14 days [3,4].

## Case presentation

An unmarried male in his mid-30s was referred to our tertiary care center from a primary healthcare facility with a positive VDRL report. On inquiry, he revealed a history of unprotected sexual exposure on one occasion nine months back which was not followed by genital ulcers or cutaneous rash. He also did not complain of headaches, photophobia, or cognitive dysfunction. No signs of meningeal irritation or cranial nerve palsy were elicited on examination.

Initial serological investigations were performed after obtaining informed consent from the patient. Combined serum investigation for human immunodeficiency virus (HIV) by chemiluminescent microparticle immune assay containing p24 antigen, HIV-1 antibodies, and HIV-2 antibodies was reactive. Only HIV-1 antibodies were detected by immunochromatography. The patient’s CD4+ T-lymphocyte count was determined to be 298 cells/µL. A cartridge-based nucleic acid amplification test for tuberculosis was negative, and the patient was started on antiretroviral therapy. Serum VDRL testing with serial dilution by flocculation method revealed reactivity with a titer of 1:128. Serum *Treponema pallidum* hemagglutination test was positive. VDRL testing of a CSF sample was reactive with a titer of 1:160.

The standard treatment regimen, as per the Centers for Disease Control and Prevention (CDC), with administration of intravenous aqueous crystalline penicillin G or intramuscular procaine penicillin G with probenecid per oral was planned. However, none of these medications were available in the city or its surrounding districts. Therefore, the patient was put on doxycycline 200 mg per oral twice daily for 28 days. Concurrently, intramuscular benzathine penicillin 2.4 million units once every week for three weeks was administered.

At follow-up after six months, the patient was still asymptomatic, and serum VDRL with serial dilution was non-reactive. However, CSF-VDRL revealed a rise in titer to a dilution of 1:320 which was suggestive of treatment failure. He was then administered ceftriaxone 1 g intramuscularly for 14 consecutive days.

The patient was again followed up in our center six months later. He remained asymptomatic during this period. His serum VDRL as well as CSF-VDRL were found to be non-reactive.

## Discussion

Considering the temporal profile from the time of sexual contact, our patient was deemed to be a case of early latent syphilis. On detection of HIV co-infection, a lumbar puncture was performed. Based on abnormal CSF findings in the absence of neurological symptoms, a diagnosis of asymptomatic neurosyphilis was made.

Due to the unavailability of first-line therapy, intramuscular ceftriaxone was offered to the patient. However, he opted to not attend the outpatient clinic for this. Treatment was then instituted with oral doxycycline which has been recommended as an alternative regimen for neurosyphilis in the current UK guidelines [4]. A recent study showed complete clinical and serological response in all cases of early neurosyphilis including those with HIV co-infection treated with oral doxycycline [5]. However, the outcome observed in our patient is at variance with the above study.

The patient was concurrently administered intramuscular benzathine penicillin, as recommended for latent syphilis. Given the rarity of symptomatic neurosyphilis in those who have not been subjected to lumbar puncture, it is likely that CSF abnormalities due to neural invasion resolve with the antibiotic regimen given for latent syphilis [1,2]. However, CSF abnormalities failed to resolve in the present case.

The patient was then counseled and administered intramuscular ceftriaxone on an outpatient basis as an alternative regimen for neurosyphilis, as recommended in the CDC treatment guidelines for sexually transmitted infections [3]. Although data on the use of ceftriaxone is limited [3], a recent study showed clinical and serological responses in 98% and 88% of neurosyphilis cases, respectively, at the end of six months which was comparable to the response with aqueous crystalline penicillin G [6].

The detection and treatment of asymptomatic neurosyphilis remains a gray area. In the latest CDC guidelines, CSF examination is not recommended in the absence of clinical signs of neurosyphilis [3]. However, concerns have been expressed that a persistent reservoir of *Treponema pallidum* in the central nervous system, though asymptomatic, may result in treatment failure as well as neurological sequelae in cases of syphilis with HIV co-infection [7]. Certain studies, as well as treatment guidelines, recommend that in asymptomatic patients with HIV co-infection, CSF examination should be performed if CD4 cell count is ≥350/mm³ and/or VDRL titer is >1:32, as was observed in our case [8,9].

## Conclusions

On failure of doxycycline as an alternative regimen for neurosyphilis in the present case, ceftriaxone was used to achieve a complete serological response. Given the sequence of events and the disease course in our patient, we conclude that ceftriaxone as an alternative regimen is still effective in the management of asymptomatic neurosyphilis even in cases with HIV co-infection.
